# Alzheimer’s disease as a multistage process: an analysis from a population-based cohort study

**DOI:** 10.18632/aging.101816

**Published:** 2019-02-27

**Authors:** Silvan Licher, Kimberly D. van der Willik, Elisabeth J. Vinke, Pinar Yilmaz, Lana Fani, Sanne B. Schagen, M. Arfan Ikram, M. Kamran Ikram

**Affiliations:** 1 Department of Epidemiology, Erasmus MC - University Medical Center Rotterdam, Rotterdam, the Netherlands; 2 Department of Psychosocial Research and Epidemiology, Netherlands Cancer Institute, Amsterdam, the Netherlands; 3 Department of Radiology and Nuclear Medicine, Erasmus MC - University Medical Center Rotterdam, Rotterdam, the Netherlands; 4 Department of Psychology, University of Amsterdam, Amsterdam, the Netherlands; 5 Department of Neurology, Erasmus MC - University Medical Center Rotterdam, Rotterdam, the Netherlands

**Keywords:** Alzheimer’s disease, genetic risk score, *APOE*, epidemiology, genetics

## Abstract

In cancer research, multistage models are used to assess the multistep process that leads to the onset of cancer. In view of biological and clinical similarities between cancer and dementia, we used these models to study Alzheimer’s disease (AD). From the population-based Rotterdam Study, we included 9,362 non-demented participants, of whom 1,124 developed AD during up to 26 years of follow-up. Under a multistage model, we regressed the logarithm of AD incidence rate against the logarithm of five-year age categories. The slope in this model reflects the number of steps (n–1) required for disease onset before the final step leading to disease manifestation. A linear relationship between log incidence rate and log age was observed, with a slope of 12.82 (95% confidence interval: 9.01-16.62), equivalent to 14 steps. We observed fewer steps for those at high genetically determined risk: 12 steps for *APOE*-ε4 carriers, and 10 steps for those at highest genetic risk based on *APOE* and a genetic risk score. The pathogenesis of AD complies with a multistage disease-model, requiring 14 steps before disease manifestation. Genetically predisposed individuals require fewer steps indicating that they already inherited multiple of these steps. Unravelling these steps in AD pathogenesis could benefit the development of intervention strategies.

## Introduction

Over the past decades, major advances have been made in the understanding of the role of amyloid and cerebrovascular pathology in the onset and progression of Alzheimer’s disease (AD) [1]. However, the underlying number of pathological changes and the subsequent final trigger leading to clinical disease manifestation, remain largely unclear. AD has a strong genetic component with a heritability of 60-80%, with additional AD-susceptibility genes that are still being identified [2,3]. These findings suggest that an individual’s genetic architecture is key in determining if and when disease emerges [2–4]. Notwithstanding the importance of environmental and lifestyle factors, it remains difficult to quantify to what extent this genetic predisposition is deterministic for AD onset.

Originated in cancer research, multistage models have been used to gain more insight in the number of steps before disease manifestation. These models are able to estimate the number of steps (‘mutations’) required for a healthy cell to become malignant [5]. After undergoing several of these rate-limiting steps, the last mutation will ultimately lead to clinical manifestation of the disease. These models have yielded consistent findings across a variety of cancers, supporting the notion that the occurrence of cancer is the end result of seven, successful mutations [5].

Cancer and neurodegenerative disease, including AD as its most common form, may be seen as two opposite ends in cell proliferation. Yet they share biological and clinical characteristics, including dysregulations in key DNA repair and inflammation processes, an increasing incidence with advancing age, and rapid disease progression after diagnosis [6,7]. Moreover, they share a complex inheritance pattern with genetic pleiotropy [8]. For instance, a recent GWAS found a positive genetic correlation between AD and cancer genes, further supporting the genetic overlap between these two diseases [8].

Given the commonalities between neurodegenerative diseases and cancer, the multistage model has recently been successfully applied to model the incidence rate of amyotrophic lateral sclerosis, a rare neurodegenerative disease, as a six-step process [9]. So far, this multistage modelling has not been used for AD. We therefore applied a multistage model within a large, population-based study to test the hypothesis that AD is a multistage process. We determined the number of steps required for disease onset and hypothesized that if AD complies with a multistage process, the number of steps will be smaller in genetically predisposed individuals as these individuals may already inherited one of these key steps.

## RESULTS

During a follow-up of up to 26.1 years, 1,124 out of 9,362 participants were diagnosed with AD, (median follow-up 10.3 years [interquartile range 10.1 years].). Table 1 shows the baseline characteristics of the study population. In this sample, 58.2% of the participants were women. Of the included participants, 2,624 were *APOE* ε4 carriers (28.0%).

**Table 1:** Baseline characteristics of total study population.

**Characteristic**	**Study population** **(N=9,362)**
Age, median (IQR), y	65.0 (12.6)
Women	5,453 (58.2)
*APOE* ・4 carrier	2,624 (28.0)
Weighted genetic risk score	
First tertile	3,146 (33.6)
Second tertile	3,120 (33.3)
Third tertile	3,096 (33.1)
Educational level	
Primary	1,689 (18.3)
Lower	3,941 (42.6)
Further	2,512 (27.2)
Higher	1,101 (11.9)
Body mass index, mean (SD), kg/m^2^	26.8 (3.9)
Systolic blood pressure, mmHg, mean (SD)	140 (22)
Diastolic blood pressure, mmHg, mean (SD)	76 (12)
Total cholesterol, mmol/L, mean (SD)	6.3 (1.2)
Diabetes mellitus	1,026 (11.0)
Smoking status	
Never	3,021 (32.7)
Former	4,276 (46.3)
Current	1,938 (21.0)
No alcohol use	1,416 (17.3)

Abbreviations: *APOE*, apolipoprotein E; IQR, interquartile range; SD, standard deviation. Data are presented as number (percentage) of participants unless otherwise indicated. Values are shown without imputation and therefore not always add up to 100%.

### Multistep model

**Figure 1:**
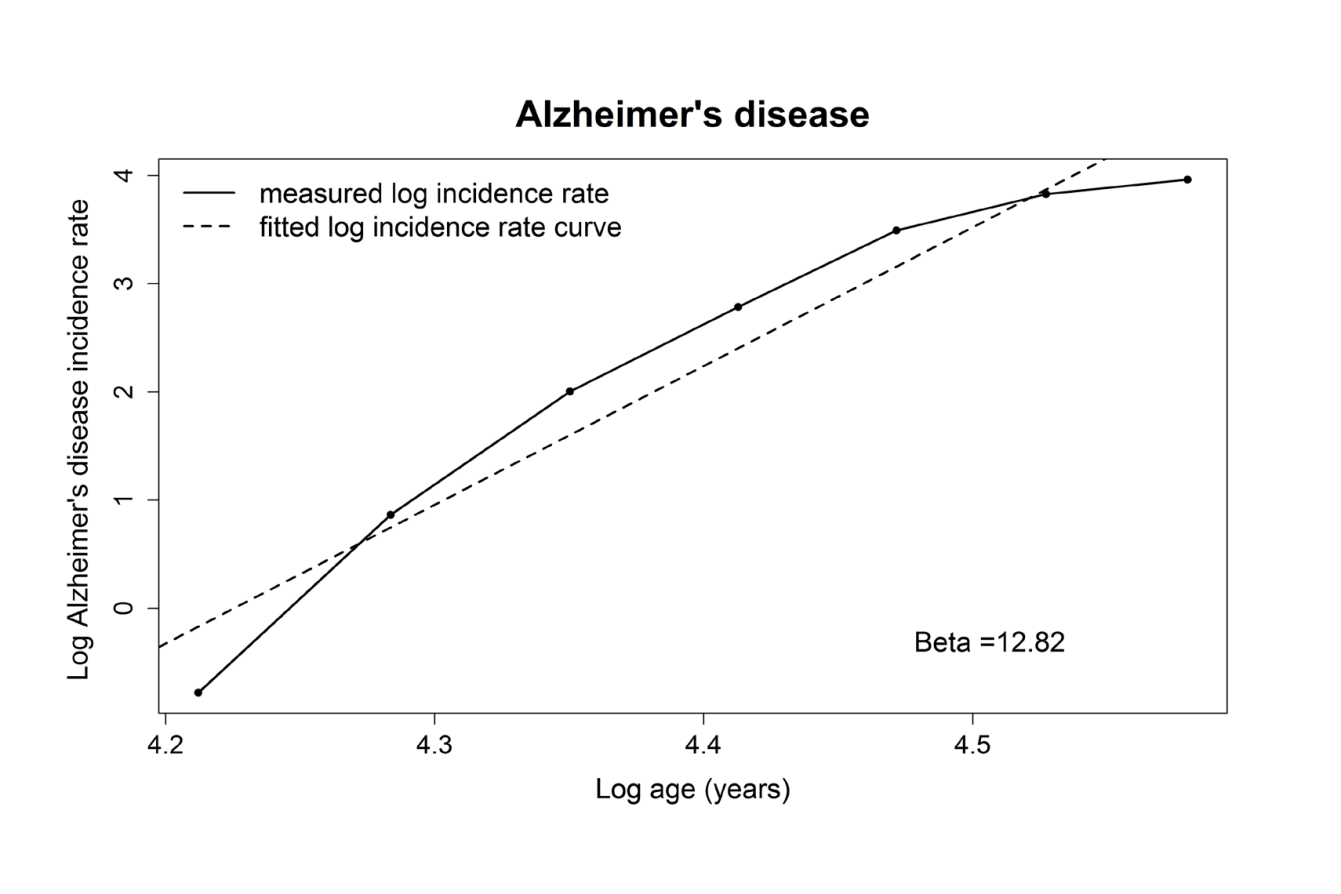
**Plotted log incidence rate of Alzheimer’s disease (y-axis) against log age (x-axis). **The dashed line shows the most optimal linear correlation.

The adjusted R-squared for the relation between log AD incidence rate and log age was 0.93, indicating a linear correlation, which is in line with the multistage model. The estimate of the slope (number of steps minus 1) for AD was 12.8 (95% confidence interval (CI): 9.0-16.6), indicating that 14 steps are needed for the development of AD (Table 2, Figure 1).

**Table 2:** Overview of estimates for slopes across groups with different genetic risks.

**Study population**		**n/N**	***n*-1** **(95%CI)**	R-squared*
Total study population		1,124/9,362	12.82 (9.01-16.62)	0.925
				
*APOE* ・4	Carrier	481/2,624	10.56 (5.96-15.17)	0.849
	Homozygote	70/213	8.92 (5.74-12.11)	0.923
	Heterozygote	411/2,411	14.93 (8.11-21.75)	0.878
	Non-carrier	643/6,738	15.02 (11.28-18.76)	0.946
				
Weighted genetic risk score tertile	First	296/3,146	15.04 (9.41-20.68)	0.885
	Second	376/3,120	12.8 (9.94-15.65)	0.956
	Third	452/3,096	11.72 (7.37-16.08)	0.886
				
Weighted genetic risk score first tertile	*APOE* ・4 carrier	124/843	8.47 (1.91-15.04)	0.886
	*APOE* ・4 non-carrier	172/2,303	15.3 (11.77-18.82)	0.954
Weighted genetic risk score second tertile	*APOE* ・4 carrier	161/930	10.31 (6.83-13.79)	0.905
	*APOE* ・4 non-carrier	215/2,190	15.54 (12.02-19.05)	0.955
Weighted genetic risk score third tertile	*APOE* ・4 carrier	196/851	8.93 (3.51-14.36)	0.738
	*APOE* ・4 non-carrier	256/2,245	14.39 (9.81-18.97)	0.915

Abbreviations: *APOE*, apolipoprotein E; n, number of incident Alzheimer’s disease events; N, total number of participants; *n*-1, estimate for slope (i.e. number of steps minus 1).

*Obtained from linear regression model log(Alzheimer’s disease incidence_i_) = β_0_ + β_1_*log(age_i_)

### Considering genetic risk

When considering only the *APOE*-related risk of developing AD, we found that *APOE* ε4 genotype non-carriers needed more steps to develop AD compared to *APOE* ε4 carriers (16 steps for non-carriers, 12 for carriers). In an exploratory analysis, we also examined the number of steps among participants homo- or heterozygous for *APOE* ε4 separately. Participants homozygous for the *APOE* ε4 allele required 10 steps, while participants heterozygous for *APOE* with ε3 and ε4 or ε2 and ε4 required 16 steps to develop AD. Similarly, we found for participants in the low-risk tertile of the genetic risk score that more steps were required to develop AD compared to those in the high-risk tertile (16 steps versus 13 steps). When stratifying on both *APOE* ε4 carrier ship and the genetic risk score, we found that for every increase in tertile of the genetic risk score, *APOE* ε4 carriers needed less steps to develop AD compared to the *APOE* ε4 non-carriers. This translated into ten steps for *APOE* ε4 carriers in the high-risk tertile, compared to 16 steps for non-carriers for *APOE* in the low-risk tertile (Table 2).

## DISCUSSION

In this population-based study using long-term follow-up of AD, we found evidence that the development of AD follows a multistage process with 14 steps. This indicates that 14 steps are required for the clinical occurrence of AD in the general population. The number of steps was modified by the level of genetic predisposition, translating into six less steps for those individuals at highest genetic risk for AD, compared to those at the lowest genetic risk.

The multistage models have been extensively used in cancer research to provide more insight in their underlying pathogenesis [10–14]. Several studies showed that seven steps were required to develop cancer, which may reflect somatic mutations, genomic rearrangements, or changes in tissue interactions and environment. Neurodegenerative diseases show several similarities with cancer such as dysregulation of DNA repair mechanisms. Yet, the multistage model has only been applied to amyotrophic lateral sclerosis which appears to follow a multistage process with six rate-limiting steps. In this study, we show that AD also can also be modelled as a multistage condition consisting of 14 steps, stressing the genetic complexity and the variety of potential biological pathways involved in the development of this disease.

We found that the number of steps for AD differed between individuals with different degrees of genetic predisposition. *APOE* ε4 carriers require a smaller number of steps to develop AD compared to *APOE* ε4 non-carriers. Moreover, these effects became even more pronounced when additionally considering 23 AD-associated genetic variants. Compared to those at highest genetic risk (i.e. *APOE* ε4 carrier and within the third tertile of the weighted genetic score), individuals at lowest genetic risk (i.e. *APOE* ε4 non-carrier and within the first tertile of weighted genetic score) needed six more rate-limiting steps to develop AD. These findings are in line with previous observations in cancer research showing different thresholds before disease becomes clinically apparent between inherited and sporadic cancer events. For instance, individuals with familial adenomatous polyposis are at increased risk of colon cancer due to one mutated copy of the *APC* gene. It has been shown that these individuals need one step fewer in the overall pathological process to develop clinical colon cancer than individuals without this mutated gene [10]. Furthermore, children with inherited retinoblastoma required only one hit to develop this disease, whilst sporadic retinoblastoma cases became clinically apparent after two hits [15]. Our findings may suggest that individuals with genetic predisposition begin several stages further down the chain of the required pathological threshold before AD becomes clinically apparent.

Although our findings suggest that 14 steps are needed for AD to emerge clinically, the underlying biological pathways and changes reflected by these steps still need to be identified. To date, eight different biological pathways involved in the pathogenesis of AD have been identified using genetic variants in AD [16]. The *APOE* ε4 allele is the most significant genetic risk factor due to its high prevalence and strong relation to AD. It is involved in four of these pathways, including cholesterol transport, hematopoietic cell lineage, clathrin/AP2 adaptor complex, and protein folding pathways. Our finding that *APOE* ε4 non-carriers need four more steps before AD clinically manifests compared to *APOE* ε4 carriers taps into this observation, and could indicate that changes in the abovementioned four pathways are indeed necessary to acquire before AD manifests clinically. This could mean that these pathways are already changed or dysregulated at birth in *APOE* ε4 carriers, indicating that these individuals subsequently have a lower resilience to the development of dementia. This could in turn lead to a lower required number of subsequent steps before disease manifestation. Indeed, up to 18% of the *APOE* ε4 carriers in this study developed AD during follow-up, yet the lifetime risk of AD among these individuals is even higher with almost half of all them developing AD in their remaining lifetime. For carriers homozygous for *APOE* ε4 in the high-risk tertile, this risk is even higher, and the disease moreover manifests earlier, with a 29-year difference in age at onset for AD, compared to homozygous *APOE* carriers at the low-risk tertile of the genetic risk score [2].

The search of finding successful AD therapies is among the most challenging and expensive healthcare issue to date. So far, many disease-modifying agents aim to reduce the production of amyloid-beta (Aβ), or target a specific but single part of the disease process [17]. Our present study shows that as many as 14 steps are required before AD becomes clinically apparent. This high number of required steps may signal the need to develop multi-domain approaches to target various underlying disease-processes simultaneously in order to halt or deter neurodegeneration.

Several limitations of this study need to be discussed. Firstly, although the use of multistage models produces a number as simple, and concrete result, its exact biological meaning is complex and remains hard to interpret. For instance, multistage models reflect the notion and the trajectory of a single cell or cell lineage to become malignant in several rate limiting steps in cancer research. However, the biological unit and meaning of these independent steps is more variable in the case of AD, as indeed it is for other neurodegenerative diseases such as ALS. This could for instance reflect an essential pathophysiological change in a single neurovascular unit, but could also relate to a key genetic mutation in a single cell or cell lineage. Secondly, the underlying multistage model assumes that disease development is predominantly genetically determined. This means that a certain number of steps, all with a similar exposure time, have to occur before the specific disease manifests clinically. In most instances, this means that the exposure under study must be present at birth or during an individual’s early life, such as their genes, ethnicity, sex or environmental factors present from birth onwards. This leaves little room for the incorporation of environmental factors that start later in life, such as smoking. While AD has a strong genetic component [2]. the importance of lifestyle and environmental factors is also substantial [18,19]. These factors remain however in part unaddressed in the current multistage models. Some studies in cancer epidemiology have tried to model these effects in more complex multistage models, but the results of these models turned out to be difficult to interpret and are currently poorly validated [10]. Since this is the first application of the multistage modelling in AD, we relied on a more simple, yet widely used multistage model. Future research is encouraged to incorporate (time-varying) extensions with environmental and lifestyle factors. Thirdly, results derived from exploratory analyses amongst participants either homo- or heterozygous for *APOE* ε4 should be interpreted with caution as these analyses are based on relatively small sample sizes. Fourthly, due to various reasons including for instance selection bias, the presented frequencies of homo- and heterozygous carriers for the *APOE* ε4 allele in this population-based cohort study (2.8% homozygous, 25.8% heterozygous), may differ from those in the unselected general population [20]. Nevertheless, the frequencies in this study fell within the reported ranges from several other, large population-based cohort studies (Supplementary Table 1). Finally, estimates of multistage models are vulnerable for several artificial influences on the observed incidence patterns, such as community-wide disease screening programs or misclassification of diagnoses at high ages due to restrained diagnostic work-ups [21]. For some diseases, this subsequently could influence the estimation of the slope and thus the number of steps needed for disease onset. We nevertheless minimized these effects by using a cohort study with standardized and consistent AD ascertainment over time with virtually complete follow-up (>95% of potential person-years).

In conclusion, we found that AD complies with a multistage model characterized by 14 steps that include essential facets of biological change which are required before AD becomes clinically apparent. Moreover, we observed that individuals with a higher genetic susceptibility require less of these additional steps before disease manifests clinically. Future research is warranted to validate the number of steps, to study the effects of environmental and lifestyle factors, and to further investigate the processes underlying these rate-limiting steps. These findings could further increase the understanding of the pathogenesis of AD, which in turn could benefit the development of prevention and treatment strategies.

## MATERIALS AND METHODS

### Study design

This study was embedded within the Rotterdam Study, a prospective population-based cohort designed to study the occurrence and determinants of age-related diseases in the general population. Details regarding the objectives and design have been reported previously [22]. Briefly, in 1990 inhabitants aged ≥55 years from a well-defined suburb in the city of Rotterdam, the Netherlands were invited to participate. The initial cohort comprised 7,983 individuals. In 2000, 3,011 individuals who had become 55 years of age or moved into the study district since the start of the study if aged ≥55 years, were added to the cohort. In 2006, a further extension of the cohort was initiated in which 3,932 individuals were included, aged ≥45 years. In total, the Rotterdam Study comprises 14,926 individuals aged ≥45 years. The overall response rate for all three recruitment waves was 72%.

This study is registered with the Netherlands National Trial Register and WHO International Clinical Trials Registry Platform under the shared catalogue number NTR6831. The Rotterdam Study was approved by the medical ethics committee of the Erasmus MC University Medical Center Rotterdam (registration number MEC 02.1015) and by the Dutch Ministry of Health, Welfare and Sport (Population Screening Act WBO, license number 1071272-159521-PG). Written informed consent was obtained for all participants. This study is registered with the Netherlands National Trial Register and WHO International Clinical Trials Registry Platform under the shared catalogue number NTR6831.

**Figure 2:**
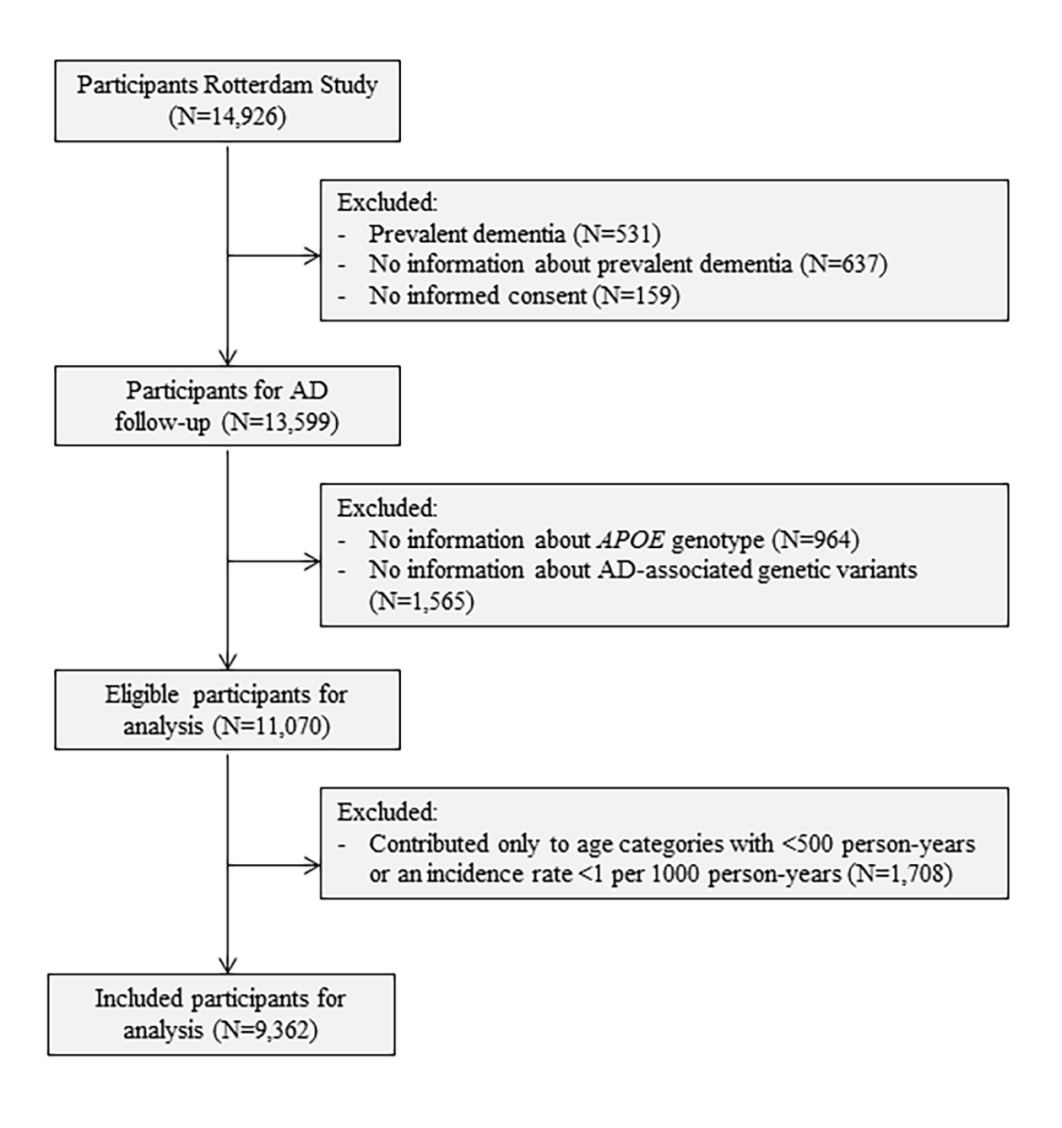
**Flowchart of study population.** Abbreviations: AD, Alzheimer’s disease; *APOE*, Apolipoprotein E.

To model AD as a multistage process, we excluded participants with a history of any type of dementia at baseline (N=531) and those who were insufficiently screened for dementia (N=637). We further excluded participants who did not provide informed consent to access medical records or hospital discharge letters (N=159). Lastly, participants without information on their *APOE* genotype (N=964) or AD-associated genetic variants to calculate the genetic risk score (N=1,565) were excluded, leaving 11,070 participants for analyses (Figure 2).

### APOE genotyping and calculation of a weighted genetic risk score

DNA was extracted from blood samples drawn by venepuncture at baseline. *APOE* genotype was determined using polymerase chain reaction on coded DNA samples in the initial cohort and with a bi-allelic TaqMan assay (rs7412 and rs429358) in the two extensions (RS-II and RS-III). The majority of samples (81.1%) were further genotyped with the Illumina 610K and 660K chips and imputed to the Haplotype Reference Consortium reference panel (version 1.0) with Minimac 3. We included 23 genetic variants that showed genome wide significant evidence of association with AD to calculate a weighted genetic risk score (Supplementary Table 2 for an overview of the included variants) [9,23–37]. This score was calculated as the sum of the products of single nucleotide polymorphism dosages of the 23 genetic variants (excluding *APOE*) and their respective reported effect estimates. All 23 variants selected for the calculation of the genetic risk score were well imputed (imputation score R2 > 0.3, median 0.99).

### Ascertainment methods of dementia

Baseline and follow-up ascertainment methods for dementia have previously been described in detail [19]. Participants were screened for dementia at baseline and subsequent centre visits with the Mini-Mental State Examination and the Geriatric Mental Schedule organic level. Those with a Mini-Mental State Examination score <26 or Geriatric Mental Schedule score >0 underwent further investigation and informant interview, including the Cambridge Examination for Mental Disorders of the Elderly. All participants also underwent routine cognitive assessment. In addition, the entire cohort was continuously under surveillance for dementia through electronic linkage of the study database with medical records from general practitioners and the regional institute for outpatient mental health care. Available information on cognitive testing and clinical neuroimaging was used when required for diagnosis of dementia subtype. A consensus panel led by a consultant neurologist established the final diagnosis according to standard criteria for AD (NINCDS–ADRDA). Participants were censored at date of any type of dementia diagnosis, death, loss to follow-up, or 1st January 2016, whichever came first. Follow-up was virtually complete (96.3% of potential person-years) [38].

### The multistage model

Multistage models originate from cancer epidemiology, where they were first employed to study the age distribution of several cancer types [5,12,39,40]. Within this framework it is assumed that cancer manifests clinically after a certain threshold number has been reached composed of *n* mutations within one cell. This threshold for disease occurrence in that cell has a certain probability distribution over time *(t)*, e.g. for an individual the *n^th^* mutation occurs at age 50, whereas for another individual this *n^th^* mutation may occur at age 80. Of the required mutations, *(n*-1*)* mutations have independently taken place at a certain point during the lifespan. For each of these mutations, a certain probability per time unit (e.g., year) exists that a mutation will occur (λ). When a cell is primed, such that it has undergone all of these necessary preceding mutations, the final mutation (*n^th^* mutation) leads to clinical manifestation of disease. Subsequently, this final *n^th^* mutation has to occur after all of these steps and can for example not occur in between preceding steps. So, the probability density function of time-point *t*, when the n^th^ change takes place is:

$$f\left(t\right)\;\sim \;\lambda_{1}\lambda_{2}\ldots \lambda_{n-1}\lambda_{n}t^{n-1}$$

It was noted in cancer epidemiology that the age-specific incidence rate of cancer *(‘i’)* roughly coincided with the probability that at least one cell of all independent cells acquired the necessary number of seven mutations by that specific age. This means that for most types of cancer six preceding rate-limiting steps *(n*-1*)* are necessary during the lifespan, with a seventh and final mutation (*n^th^* mutation), leading to disease manifestation [41]. It can subsequently be shown that if the disease under study fits a multistep process, the number of these steps $\left(n\right)$ can be estimated with the following formula:

$$\log\left(i\right)=\left(n-1\right)\log\left(t\right)+c$$

in which c is a constant number containing $log(\lambda_{1}\lambda_{2}\ldots \lambda_{n-1}\lambda_{n})$. The common ground of these rate-limiting definitions is that the speed of a reaction step will have a significant effect on the speed of the overall chain of events to which the step belongs [42]. A reaction step is thus subsequently considered a rate-limiting step, when the rate of that particular step is identical to the overall rate of the entire reaction.

### Statistical analysis

We applied a multistage model to determine the slope and the number of steps for the development and clinical onset of AD. In line with previous studies, the incidence rate of AD was calculated per five years age categories [5,9]. Each participant contributed person-years to specific age categories, until the age at AD diagnosis or censoring. To minimize the effects of outliers on the slope of the model, we excluded age categories with less than 500 person-years or with an incidence rate below 1 per 1000 person-years given that estimated incidence rates often become instable in the extremes of the age distribution [40]. This additional criterion resulted in an exclusion of 213,530.6 person-years, which corresponded to the exclusion of 1,708 of the 11,070 participants with age at AD or censoring below the first included age category. This left 9,362 participants available for the final analyses (Figure 2). The incidence rate of AD and the five-years age categories (log age) were natural log-transformed. Linearity was tested based on the adjusted R-squared obtained from a linear regression model with log age and incidence rate of AD as outcome. Linear models were unadjusted.

Additionally, we stratified according to *APOE* ε4 carrier status and on tertiles of a weighted genetic risk score in mutually exclusive categories of genetic risk and by combining both in order to be able to stratify those individuals with the lowest and those with the highest AD genetic risk.

Data were handled and analysed with SPSS Statistics version 24.0.0.1 (IBM Corp., Armonk, NY) and R, CRAN version 3.4.3.

## SUPPLEMENTARY MATERIALS

Supplementary Tables
