# Supplementary material for: Alzheimer’s disease as a multistage process: an analysis from a population-based cohort study
Source: Aging (Albany NY). 2019 Feb 27;11(4):1163–76. doi: 10.18632/aging.101816 (PMC6402512; doi:10.18632/aging.101816)
Supplement: Supplementary Tables [file aging-11-101816-s001.pdf]

SUPPLEMENTARY MATERIAL

Supplementary Table 1. Frequency of *APOE* alleles in study population.

***APOE* genotype**	**Study population (N=9,362)**
ε2/ε2	63 (0.7)
ε2/ε3	1,208 (12.9)
ε2/ε4	250 (2.67)
ε3/ε3	5,467 (58.4)
ε3/ε4	2,161 (23.1)
ε4/ε4	213 (2.8)

Abbreviations: *APOE*, apolipoprotein E.
Data are presented as number (percentage) of participants.

Supplementary Table 2. Genetic variants included in the genetic risk score.

Chr	Rs-id	ALT-HRC	Assigned-gene	Locus discovered in:	Effect estimate	Maf	Weight ALT-HRC	R^2-RS1	R^2-RS2	R^2-RS3
19	rs4147929	G	*ABCA7*	Hollingworth et al. , Naj et al.	Lambert et al. (2013)	0.19	-0.135	0.916	0.917	0.991
2	rs6733839	T	*BIN1*	Seshadri et al.	Lambert et al. (2013)	0.409	0.188	0.960	0.911	0.962
20	rs7274581	C	*CASS4*	Lambert et al. (2013)	Lambert et al. (2013)	0.083	-0.139	0.990	0.989	0.990
6	rs10948363	G	*CD2AP*	Hollingworth et al. , Naj et al.	Lambert et al. (2013)	0.266	0.098	0.998	0.998	0.998
11	rs10838725	C	*CELF1*	Lambert et al. (2013)	Lambert et al. (2013)	0.316	0.075	0.998	0.998	0.998
8	rs9331896	T	*CLU*	Harold et al. , Lambert et al. (2009)	Lambert et al. (2013)	0.379	0.146	0.902	0.974	0.901
1	rs6656401	G	*CR1*	Lambert et al. (2009)	Lambert et al. (2013)	0.197	-0.157	0.953	0.948	0.950
10	rs7920721	G	*ECHDC3*	Desikan et al.	Desikan et al.	0.387	-0.067	1.000	1.000	1.000
7	rs11771145	A	*EPHA1*	Hollingworth et al. , Naj et al.	Lambert et al. (2013)	0.338	-0.102	0.998	0.998	0.999
14	rs17125944	C	*FERMT2*	Lambert et al. (2013)	Lambert et al. (2013)	0.092	0.122	1.000	1.000	1.000
6	rs111418223	A	*HLA-DRB1/5*	Lambert et al. (2013)	Lambert et al. (2013)	0.276	-0.108	0.314	0.312	0.314
4	rs13113697	G	*HS3ST1*	Desikan et al.	Desikan et al.	0.283	-0.067	0.999	0.998	0.999
2	rs35349669	T	*INPP5D*	Lambert et al.	Lambert et al. (2013)	0.488	0.066	0.975	0.973	0.976
17	rs118172952	G	*KANSL1*	Jun et al.	Lambert et al. (2013)	0.873	-0.151	0.710	0.700	0.708
5	rs190982	A	*MEF2C*	Lambert et al. (2013)	Lambert et al. (2013)	0.408	0.080	0.979	0.934	0.978
11	rs983392	G	*MS4A6A*	Hollingworth et al. , Naj et al.	Lambert et al. (2013)	0.403	-0.108	0.989	0.990	0.991
7	rs2718058	G	*NME8*	Lambert et al. (2013)	Lambert et al. (2013)	0.373	-0.070	1.000	1.000	1.000
11	rs10792832	G	*PICALM*	Harold et al.	Lambert et al. (2013)	0.358	0.130	0.999	0.999	0.999
8	rs28834970	C	*PTK2B*	Lambert et al. (2013)	Lambert et al. (2013)	0.366	0.096	0.993	0.990	0.994
14	rs10498633	T	*SLC24A4-RIN3*	Lambert et al. (2013)	Lambert et al. (2013)	0.217	-0.104	0.999	0.999	1.000
11	rs11218343	C	*SORL1*	Lambert et al. (2013)	Lambert et al. (2013)	0.039	-0.270	0.998	0.995	0.998
6	rs75932628	T	*TREM2*	Guerreiro et al. , Jonsson et al.	Ruiz et al.	0.0016	0.889	0.762	0.726	0.668
7	rs1476679	T	*ZCWPW1*	Lambert et al. (2013)	Lambert et al. (2013)	0.287	0.078	0.995	0.996	0.995

Ordered by assigned gene name. References: Harold et al.[1], Seshadri et al.[2], Hollingworth et al.[3], Naj et al.[4], Lambert et al. (2009,2013)[5, 6], Jonsson et al.[7], and Ruiz et al.[8] Minor allele Frequency (Maf) of Rotterdam Study (RS) 1 is shown and is representative of the MAF in RS2 and RS3. R^{2}= imputation quality. RS1= initial Rotterdam Study cohort, RS2=first extension Rotterdam Study, RS3=second extension. Rotterdam Study cohorts were imputed separately. Gene names are ncbi gene names assigned to the loci in the corresponding references.

SUPPLEMENTARY REFERENCES

1. Harold D, Abraham R, Hollingworth P, Sims R, Gerrish A, Hamshere ML, Pahwa JS, Moskvina V, Dowzell K, Williams A, Jones N, Thomas C, Stretton A, et al. Genome-wide association study identifies variants at CLU and PICALM associated with Alzheimer's disease. Nat Genet. 2009; 41:1088–93. https://doi.org/10.1038/ng.440

2. Seshadri S, Fitzpatrick AL, Ikram MA, DeStefano AL, Gudnason V, Boada M, Bis JC, Smith AV, Carassquillo MM, Lambert JC, Harold D, Schrijvers EM, Ramirez-Lorca R, et al, and CHARGE Consortium, and GERAD1 Consortium, and EADI1 Consortium. Genome-wide analysis of genetic loci associated with Alzheimer disease. JAMA. 2010; 303:1832–40. https://doi.org/10.1001/jama.2010.574

3. Hollingworth P, Harold D, Sims R, Gerrish A, Lambert JC, Carrasquillo MM, Abraham R, Hamshere ML, Pahwa JS, Moskvina V, Dowzell K, Jones N, Stretton A, et al, and Alzheimer's Disease Neuroimaging Initiative, and CHARGE consortium, and EADI1 consortium. Common variants at ABCA7, MS4A6A/MS4A4E, EPHA1, CD33 and CD2AP are associated with Alzheimer's disease. Nat Genet. 2011; 43:429–35. https://doi.org/10.1038/ng.803

4. Naj AC, Jun G, Beecham GW, Wang LS, Vardarajan BN, Buros J, Gallins PJ, Buxbaum JD, Jarvik GP, Crane PK, Larson EB, Bird TD, Boeve BF, et al. Common variants at MS4A4/MS4A6E, CD2AP, CD33 and EPHA1 are associated with late-onset Alzheimer's disease. Nat Genet. 2011; 43:436–41. https://doi.org/10.1038/ng.801

5. Lambert JC, Heath S, Even G, Campion D, Sleegers K, Hiltunen M, Combarros O, Zelenika D, Bullido MJ, Tavernier B, Letenneur L, Bettens K, Berr C, et al, and European Alzheimer's Disease Initiative Investigators. Genome-wide association study identifies variants at CLU and CR1 associated with Alzheimer's disease. Nat Genet. 2009; 41:1094–99. https://doi.org/10.1038/ng.439

6. Lambert JC, Ibrahim-Verbaas CA, Harold D, Naj AC, Sims R, Bellenguez C, DeStafano AL, Bis JC, Beecham GW, Grenier-Boley B, Russo G, Thorton-Wells TA, Jones N, et al, and Cohorts for Heart and Aging Research in Genomic Epidemiology. Meta-analysis of 74,046 individuals identifies 11 new susceptibility loci for Alzheimer's disease. Nat Genet. 2013; 45:1452–58. https://doi.org/10.1038/ng.2802

7. Jonsson T, Stefansson H, Steinberg S, Jonsdottir I, Jonsson PV, Snaedal J, Bjornsson S, Huttenlocher J, Levey AI, Lah JJ, Rujescu D, Hampel H, Giegling I, et al. Variant of TREM2 associated with the risk of Alzheimer's disease. N Engl J Med. 2013; 368:107–16. https://doi.org/10.1056/NEJMoa1211103

8. Ruiz A, Dols-Icardo O, Bullido MJ, Pastor P, Rodríguez-Rodríguez E, López de Munain A, de Pancorbo MM, Pérez-Tur J, Alvarez V, Antonell A, López-Arrieta J, Hernández I, Tárraga L, et al, and dementia genetic Spanish consortium (DEGESCO). Assessing the role of the TREM2 p.R47H variant as a risk factor for Alzheimer's disease and frontotemporal dementia. Neurobiol Aging. 2014; 35:444.e1–4. https://doi.org/10.1016/j.neurobiolaging.2013.08.011
